# American Academy of Dental Science

**Published:** 1892-09

**Authors:** 

**Affiliations:** American Academy of Dental Science


### AMERICAN ACADEMY OF DENTAL SCIENCE.

THE regular monthly meeting of the American Academy of Dental Science was held at the Boston Medical Library Association Rooms, on Wednesday, April 6, 1892, at 7.30 P.M., President Brackett in the chair.

Papers were read as follows: By W. Xavier Sudduth, M.D., D.D.S.; subject, "Dental Education."

(For Dr. Sudduth's paper, see page 645.)

By Charles H. Taft, D.M.D.; subject, "Silica: Its Curative Action in the Treatment of Alveolar Abscess."

(For Dr. Taft's paper, see page 653.)

### DISCUSSION OF DR. SUDDUTH'S PAPER.

*President Brackett.*—Gentlemen, this paper is before you for discussion. It is plainly the work of an earnest man, who has the courage of his convictions, and he presents strong arguments in support of his ideas. The matter of dental education has excited much interest in this community, owing to what has been considered the somewhat peculiar position of other States.

*Dr. Fillebrown.*—My own position in this matter, and, I believe, also that of the Faculty and alumni of the Harvard Dental School, coincides with that of Dr. Sudduth. There is, therefore, no room for discussion. I heartily commend the paper, and wish that every State in the Union had a law like Massachusetts, requiring that every dentist who wishes to practise should pass an examination.

*Dr. Andrews.*—I agree with Professor Fillebrown, that the paper is so sensible in every way that there is very little to discuss. One thing I would like to say, and that is, I believe thoroughly in "every tub standing on its own bottom." We know that the graduates of certain colleges are allowed to practise abroad without examination, while the graduates of others are not. Let a system of universal examination be brought into use, and fairness and justice will be done to all. In some of the States dental colleges are owned by their professors, and are run for the profit they can get out of them.

Much of the opposition to the ruling of State law and State examinations has come from colleges whose Faculties are deeply interested in the profits of the institution. I believe heartily in the sentiment of the essayist.

The board of State examiners should, however, have some respect for what the dental colleges have done. A case occurred in the Boston Dental College where a student was rejected by the Faculty, but passed by the State examining board.

*Dr. Grant.*—We as dentists ought to exert our influence to settle the proper relations which should exist between the dental colleges and the State examining boards. The dental colleges of a State should go before the Legislature and ask that they might appoint an examining board to determine the qualifications of candidates for graduation in the dental schools, with the power of granting licenses to such graduates and others who may be qualified to practise.

There are many instances where a feeling of general bitterness has been expressed towards a dental school by an unsuccessful candidate for graduation, because he thought his rejection was unjust, being influenced in a certain degree by personal feeling on the part of some member of the board of instruction in the school. If an independent board of examiners passed upon the qualifications of all candidates no such feeling could exist. Where the executive of a State is left to appoint an examining board simply from personal acquaintance or the recommendation of another man, it is no wonder that often the proper persons are not appointed.

*Dr. Cooke.*—Considering the history of our dental law, it is a wonder to me that we have one at all; it is extremely fortunate, in my opinion, that the whole matter of dental legislation was taken out of our dental societies and put where it is. If, instead of one operation, a candidate were required to do five or six before the State board, there would be a better opportunity to determine his ability.

*Dr. Banfield.*—It seems to me very inexpedient to examine and pass a man on one simple operation.

*Dr. Andrews.*—The State board should have the State society back of them for advice and reference. The Massachusetts Dental Society should attend to this.

*Dr. Fillebrown.*—I am in full sympathy with the board, and believe thoroughly in the institution. If I criticise their actions, it is done merely to urge to higher standards.

DISCUSSION OF DR. TAFT'S PAPER.

*Dr. Hitchcock.*—If I understood the essayist correctly, he began treatment in February, 1891, and ended in March, 1892. It seems to me that if the abscess was not cured in less time than that, silica had little to do with it. I would like to ask Dr. Taft whether he purchased his peroxide of hydrogen or prepared it himself.

*Dr. Taft.*—I purchased it.

*Dr. Hitchcock.*—If I remember correctly, peroxide of hydrogen is dormant at a temperature below 40° F., and at a temperature of over 70° it loses its oxygen. The solution should be made by a competent chemist, and kept in a cool place.

*Dr. Williams.*—Dr. Taft's mention of the use of silica reminds me of a case I had fifteen or twenty years ago. It was a very obstinate ulceration, occurring after the extraction of a tooth. The patient was not in very good health, and suppuration continued for some time. I tried various remedies without effect. Dr. Cabot, who was an excellent surgeon, gave me a hint. It was to apply silicate of soda, a little diluted, perhaps half or two-thirds strength, on a bit of cotton. That seemed to have a very good result, and I applied it once a day for several days, and I think in the course of ten days the case was healed. I have found it very useful in several cases since.

*Dr. Cooke.*—I would like to ask Dr. Taft what was the strength of the dose he used in the last case.

*Dr. Taft.*—The medicine I last gave was in the one-hundred-thousandth potency.

*Dr. Cooke.*—I do not understand what virtue there can be in a solution made from the hundred-thousandth potency.

*Dr. Taft.*—I hope the gentleman does not confound a simple dilution with a potency. If you put a couple of drops of a drug in a bucket of water you get a solution with no value as a medicine; but if you take two drops of that drug and add ninety-eight drops of alcohol, and shake it, then you get a potency; then take two drops of that and add to it ninety-eight more of alcohol, and shake again, and so on,—in that way you evolve the medicinal properties of a drug.

*Dr. Williams.*—This shaking must be done by hand, as I understand it. Wouldn't it have the same effect if the solution were put in a machine and shaken up?

*Dr. Taft.*—I should think the effect would be the same. I don't know about that.

*Dr. Allen.*—I have no experience in administering homœopathic remedies in dentistry; but, if you wlll allow me, I would like to speak of two cases of their use which interested me a great deal. Each case happened to be in the family of a homœopathic physician, both physicians being eminent in their profession. One case was a chronic alveolar abscess, which originated in a central incisor that had a Richmond crown on it. The crown had been placed there by a dentist who is well known to most of us, and there is reason to believe that he had thoroughly prepared the root, and had filled it properly. Notwithstanding that, an abscess formed, followed by a fistula and a copious discharge of pus, which condition seemed to indicate the removal of the crown and further treatment of the root. I suggested to the doctor that he try his homœopathic remedies on it,—certainly a safe thing to do, you would say,—and that the dentist should not interfere until it became imperative that he should do so. My suggestion was followed, and the result was eminently satisfactory. I had occasion to examine the lady's mouth afterwards, and the apparent healthy condition of the root fully warranted the course that had been pursued.

The other case was an acute alveolar abscess in the left superior second bicuspid, which also had a crown upon it. The lady's husband was a physician, and an enthusiast in homœopathy. He refused to have the dentist treat the case, saying that he would take care of it himself. As there was very great swelling and much fever present, I did not believe that speedy relief could be obtained without lancing the abscess, nor a cure effected without opening the root. The abscess, however, was not lanced, but homœopathic remedies were administered internally, and relief followed within a very few hours, together with a rapid subsidence of the swelling.

If it is not irrelevant to the subject, I would like to give a personal experience in regard to homœopathic medicines. It was my ill fortune several years ago to injure the instep of my right foot. I was in a lecture-room, and a number of students were seated on a settee directly in front of me. They were leaning back, and finally tipped over, and I received the weight of that row of students on my instep. The injury seemed trifling at the time, but next morning my foot was very lame, and for several days continued to be so, when, happening to mention my accident to a friend who was a homœopathic physician, he said, "Let me prescribe for it,—I will give you some arnica." Said I, "Yes, I think arnica would be very good to rub my foot with." He replied, "You must take it internally." I laughed, and had no faith whatever in it, but took his dose, and next morning my foot was very comfortable, and continued so for several days, when the trouble again appeared. To make a long story short, the trouble would subside, and then reappear at intervals of a few days, the relief always being coincident with a dose of medicine administered internally. After the absence of six months, during which time the trouble again appeared, I went to him for an examination. He said the periosteum had been bruised, and that a bone abscess was threatening. At my request he prescribed for me, and told me that later on I would know what the remedy was. Relief followed immediately, and has been permanent. In reply to my question afterwards as to what the medicine was, he said it was the one-hundred-thousandth potency of arnica.

*Dr. Williams.*—I would like to ask the gentleman if in any of these cases silica was used.

*Dr. Allen.*—I do not know. It would be very easy to find out. I know that silica is used by homœopathic physicians in cases of suppuration.

*Dr. Andrews.*—I want to say a word in appreciation of this paper. I don't think we have had a better-written one in this association for a great while, and although I am not able to discuss it, I am glad that I have heard it. The action of homœopathic remedies does not seem at all improbable to those who have prepared microscopical tissues, and have noted the effect that minute percentages of reagents have on them.

*Dr. Hitchcock.*—I do not want to be understood, Mr. President, as criticising the practice of homœopathy, as I have some faith in it. But I was surprised at the length of time required for treatment in the case described by Dr. Taft.

*Dr. Taft.*—The case was undergoing treatment during that period mentioned, but I was not using silica all that time. I would say that the effect of one dose of silica will go on in the system for as long as eight weeks. I think my mistake in the treatment of the fistula was that I produced what is called an aggravation by repeating the medicine a second time too soon. I felt sure, after I had given the other two remedies without any result, that if the silica were given in a very high power it ought to cause the flow of pus to cease entirely, and the result bore out my conclusions. It shows how beautifully the higher potencies work, and shows, too, that the very word "potency" means power, and the higher the potency is carried the greater the discretion to be exercised in the use of the drug.

*Dr. Eames.*—I have always understood that mischief would not be incurred if the remedy was not applicable, or, as is popularly said, "Homœopathic medicines do no harm, even if they do no good."

*Dr. Taft.*—Well, if the selected medicine is not the *similium*, and does not reach the totality of all the symptoms, it has no effect. For instance, let a person when well take a few drops of the tincture of gelseminum. If he is at all sensitive he will notice in a few minutes that his pulse has from ten to fifteen beats fewer in the minute than it had before, with a feeling of chilliness accompanying it. This will soon be followed by heat, a quickened pulse, with pains in the head, and perhaps in the back and limbs. In short, he will find that he has a fever. Soon a prickling sensation is felt, perspiration breaks out, and in a little while he is well again. Now, it is just because gelseminum produces fever symptoms when given in large doses that it cures the same symptoms when administered in small doses.

*Dr. Eames.*—Then, if one had no disease at all, it will cause these symptoms in a healthy person? If a child gets hold of a vial-full of a certain drug and takes a quantity of it, it has the effect of producing the disease in the child for which the drug is used?

*Dr Taft.*—Certainly.

*Dr. Eames.*—I can hardly see how the shaking of the drug, as given by Dr. Taft, is different from the dilution.

*Dr. Taft.*—There are a good many things that cannot be explained,—we have got to accept them as facts. We might say, for instance, that because we cannot understand how waves of a certain kind can be sent over an electric wire so that six messages can be sent from either end at the same time, that it cannot be done. We cannot understand how hydrogen and oxygen unite in certain definite, fixed proportions to form a drop of water, but we know that they do. Just how this action is obtained with drugs nobody knows, but it is a law, and a fixed law, as those who have tested it well know.

*Dr. Meriam.*—Do we understand, then, if we get a remedy that meets all the symptoms, there is no death?

*Dr. Taft.*—I do not go so far as that,—we are talking of curable cases.

*Dr. Briggs.*—I would like to ask Dr. Taft if he uses silica in all cases of alveolar abscess, as in the one case that he has cited.

*Dr. Taft.*—No, sir; by no means. I would not be understood as saying that I use silica in every form of abscess. I very often use mercuries or hepar sulphur, the administration of either one depending entirely upon the condition of the other symptoms. They are equally valuable with silica, providing certain symptoms are present. Silica is a very slow, deep-acting drug, and, as I have said, is suited rather to chronic than to acute cases.

*Dr. Briggs.*—It is inert, is it not, on healthy human organism?

*Dr. Taft.*—Not in its medicinal form. In its crude form it is practically inert.

*Dr. Briggs.*—What are the symptoms that occur in the healthy individual in its medicinal form?

*Dr. Taft.*—It affects the whole system, from the top of the head to the tip of the toe.

*Dr. Briggs.*—Does it produce suppuration?

*Dr. Taft.*—Yes, in its physiological action.

*Dr. Briggs.*—Does Dr. Taft know how many times that has been proven?

*Dr. Taft.*—I do not; but all of the symptoms that are ascribed to it are not the provings that have been obtained by its administration to any one individual, but those that have been gathered from a number of individuals.

*Dr. Williams.*—I would like to ask if it is known who is the discoverer of this remarkable effect obtained by shaking by hand.

*Dr. Smith.*—I would like to ask Dr. Taft, if he gives this silica for chronic abscesses, as he cites in his paper, why it would not be equally serviceable for an acute form of abscess to allay the inflammation before suppuration takes place?

*Dr. Taft.*—Mercuries or hepar sulphur have the power to hasten suppuration or prevent it entirely; that is to say, if the process of suppuration has begun and cannot be averted, it will be materially hastened by the administration of either.

The case in question, Mr. President, was entirely without other symptoms. The lady was one of the healthiest individuals that I have ever seen. There was not a single symptom to aid in the selection of the remedy other than what was presented in the fistula itself. The fact that I had no success in treating it by dental agents or methods, and the fact that the discharge was so profuse all the time as to be exceedingly annoying, made it very pleasant for me to have her say of her own accord that she noticed an improvement the very next day after the administration of the medicine. It shows how nicely the medicine worked. We cannot explain how it worked, but if the lady were here she would tell you there was no question but that it did.

*Dr. Meriam.*—There is one thing to be considered in connection with this case. From Dr. Taft's description I should judge that the fistula was entirely sheltered from pressure, and such are extremely difficult to heal.

*Dr. Smith.*—Dr. Taft has said that were his patient here she would tell us that he had not overstated the case. Any one who is acquainted with Dr. Taft asks no further evidence for the truth of his assertion than his plain, unsupported statement.

The paper, I think, has been an exceedingly interesting one. He has seen fit to venture into new fields and try these drugs, and he has made, under the circumstances, a creditable success of it; and, what is more, he has the courage of his convictions, and the heroism to come here and present them.

In my dental-student days we never were taught to use remedies to abort abscesses, and I doubt very much to-day if a majority of our practitioners in dentistry are in the habit of resorting to drugs of any kind to assist in treatment. I have had under my dental care some eminent physicians and surgeons, and have purposely asked them what drug they would recommend for use in the treatment of abscessed teeth, and those eminent men say, "None." The homœopathists claim that drugs have their proper effect and will cure, and I have had cases of this kind where the patients were homœopathic physicians, and while I performed the surgical part of it, they took what they considered the proper drug, and I have always got along with these in a thoroughly satisfactory manner.

We have had cases where it is claimed that Christian science cures. I have two patients who were under treatment with the best allopathic physicians for years, men who were eminent in their profession here in Boston, and after trying everything in their science, they gave them up as incurable. Those patients went to Christian science, and in two or three months' time they were well people. Naturally, these are firm believers in the Christian-science cure, and one of them who was particularly nervous is now a much better patient; in fact, she is an excellent patient. She states that she succeeds when in the chair in absenting herself from her body. And so, gentlemen, I pay my tribute of respect to the essayist, and I am inclined to try the treatment which he has outlined.

*Mr. Houghton.*—I have been under the care of homœopathic physicians for the last two years. Before that time my health had been very poor, and I was under allopathic treatment for a number of years. I first came under the doctor's care when I was about twelve years old, and it was about that time that I began to have my teeth filled with amalgam and gold fillings. I kept growing worse until about three years ago, when I first came under the treatment of a homœopathic physician. At that time I think I was taking about a dozen different kinds of drugs, but soon began to improve under homœopathic medicines. My physician felt that I was not improving as fast as I ought, and ordered my amalgam all removed, and since then there has been a rapid change for the better.

*Dr. Williams.*—The gentleman, in relating his own experience, spoke of having two kinds of fillings in his mouth,—amalgam and gold. His physician recommended the amalgam fillings to be removed, after which he noticed a marked improvement. I want to ask if any effect was attributed to the remaining gold fillings by the physician?

*Mr. Houghton.*—Not that I know of.

*Dr. Taft.*—In closing I would say, Mr. President, that I had no intention, in presenting my paper to-night, to antagonize in any way the two great schools of medicine. It was simply to give you the benefit of the experience that I have had in administering an important and valuable drug, and incidentally I have spoken of other homœopathic remedies.

I brought with me to-night a small volume on homœopathy called "Hering's Domestic Physician," which has one or two chapters devoted entirely to the affections of the teeth. Usually there is no question about the symptoms of any case, because the patient can express those symptoms quite clearly. When a case presents itself I recognize at once certain "key-note" symptoms, which suggest a given drug. I then turn at once to the chapter alluded to, and look up the symptoms coming under it. If a large number of those symptoms do not come under that drug, I look up another, and go through as quickly as I can the different drugs to see which one has the largest number of symptoms corresponding to those of the patient, and when I find what I think is the right one, I take the large *materia medica* and confirm my opinions; then, with considerable confidence, give the medicine in the very high power I have spoken of.

I have also brought with me this medicine-case. It holds enough of the more important remedies which the dentist would need for affections of the mouth and kindred troubles. It contains only a small proportion to the number of remedies that are used in the science of homœopathy.

I have just started to make a record of every case in which systemic treatment is adopted. In this record I put down the name of the patient, all of the symptoms present, the aggravation and ameliorations, and the medicine that I give, taking pains to find out from the patient afterwards what the action of the medicine has been. In a large proportion of cases relief is given at once, and in almost all within a day or two. In some I do not select the right remedy, and consequently have to give the case further study. In this way I hope in time to get a homœopathic dental *materia medica* that will be as accurate as my own practical experience can make it. I find systemic treatment just as useful in the management of an exposed pulp, which begins immediately to ache after a careful capping, as in cases where there has been continuous pain. I had such a one come to me about a month ago. A young lady suffered a great deal after a capping that I made in an inferior bicuspid. Notwithstanding the fact that I capped it very carefully, the tooth gave her considerable trouble, and for two weeks I tried capsicum plasters and all the local obtunders of pain with which we are familiar. At first they had good effect, but the pain would soon recur. I did not want to destroy the pulp of the tooth, so I took very carefully all the symptoms, and from what I could gather thought that belladonna was the indicated remedy. I gave her the medicine, but it had no effect whatever. I told her that before destroying the pulp I would like to have one more trial, and after making a further careful study of the case, prescribed mercurius, when the pain ceased almost immediately, and she has had no serious trouble with it since.

*Dr. Briggs.*—The key-note of the use of the drug is its high potency?

*Dr. Taft.*—Yes.

*Dr. Briggs.*—And don't prominent homœopaths practise only in the lower potencies?

*Dr. Taft.*—Yes, there are a great many of those. Their number is much greater than that of those who use the high potencies.

William H. Potter, D.M.D.,
*Editor American Academy of Dental Science.*

